# A Multi-Strain Oral Probiotic Improves the Balance of the Vaginal Microbiota in Women with Asymptomatic Bacterial Vaginosis: Preliminary Evidence

**DOI:** 10.3390/nu16203469

**Published:** 2024-10-14

**Authors:** Simone Filardo, Marisa Di Pietro, Paola Mastromarino, Maria Grazia Porpora, Rosa Sessa

**Affiliations:** 1 Department of Public Health and Infectious Diseases, University of Rome “Sapienza”, 00185 Rome, Italy; marisa.dipietro@uniroma1.it (M.D.P.); rosa.sessa@uniroma1.it (R.S.); 2 Department of Maternal and Child Health and Urology, University of Rome “Sapienza”, 00161 Rome, Italy; mariagrazia.porpora@uniroma1.it

**Keywords:** oral probiotic, asymptomatic bacterial vaginosis, vaginal microbiota, next-generation sequencing

## Abstract

Background/Objectives: the vaginal microbiota is known to confer protection in the genital ecosystem, due to the predominance of different *Lactobacillus* species, playing a crucial role in women’s health; alterations in the composition of the microbial communities in the vagina can be associated with the development of bacterial vaginosis (BV). Current therapy for BV involves oral or intravaginal administration of metronidazole or clindamycin, albeit the high recurrence rates suggest a need for alternative therapeutic tools, such as probiotics. Herein, the diversity and composition of vaginal microbiota in women with asymptomatic BV was investigated before and after the oral administration of a multi-strain probiotic formulation. Methods: a prospective observational pilot study with pre–post design was carried out from 1 June 2022, to 31 December 2022, on reproductive-age women with asymptomatic BV, as diagnosed via Nugent score, and matched healthy controls. The probiotic was administered to all study participants as acid-resistant oral capsules (twice daily), and a vaginal swab was collected at baseline and after 2 months of treatment, for the metagenomic analysis of 16s rDNA. Results: the diversity and richness of the vaginal microbiota in women with BV were significantly reduced after 2 months of supplementation with the oral probiotic, as evidenced by measures of α-diversity. Interestingly, some bacterial genera typically associated with dysbiosis, such as *Megasphaera* spp., were significantly decreased; whereas, at the same time, *Lactobacillus* spp. Doubled. Conclusions: our preliminary results suggest that the multi-strain oral probiotic is a beneficial treatment specifically targeting the dysbiotic vaginal microenvironment.

## 1. Introduction

The female genital tract is colonized by bacterial communities that are known to confer antimicrobial protection to the vagina and play a crucial role in health [1,2,3].

*Lactobacillus* species are the predominant bacteria in the vaginal ecosystem of healthy women. *Lactobacillus crispatus*, *Lactobacillus gasseri*, *Lactobacillus iners*, *Lactobacillus jensenii*, or other strictly anaerobic bacteria are the dominant vaginal bacterial biotypes [4]. Alterations in the types and relative proportions of the microbial species in the vagina can be associated with the development of infectious conditions, such as bacterial vaginosis (BV), aerobic vaginitis, candidiasis, and sexually transmitted infections [5,6,7,8].

The vaginal microbiome dysbiosis, referred to as BV, is a clinical condition considered the most common and enigmatic among reproductive-age women; it is characterized by unknown aetiology and has a poorly understood pathogenesis [9,10]. The global prevalence of BV is estimated to range from 23 to 29%, although most data focus on women of reproductive age. BV can also affect post-menopausal women, albeit at a lower prevalence, estimated at around 17% globally [11]. In BV, the prevalence and concentration of *Lactobacillus* species are decreased, while several opportunistic bacteria, mainly *Gardnerella vaginalis* and other anaerobes are predominant [12].

Techniques that analyze rRNA gene sequences have become powerful tools in recent years for revealing the phylogenetic diversity of microorganisms in the vaginal ecosystem and understanding community dynamics [6,13,14,15]. According to these molecular studies, women with and without BV have significantly different vaginal bacterial communities. Higher taxonomic richness and diversity are associated with BV. The microbiota composition is highly variable among subjects at a species or genus level. However, *Actinobacteria* and *Bacteroidetes* are strongly linked to BV, while *Firmicutes* are more abundant in healthy subjects. The spectrum of anaerobes detected in BV-affected women has greatly increased due to advancements in molecular techniques, with the addition of *Eggerthella*, *Megasphaera*, *Leptotrichia*, *Dialister*, and *Sneathia* [16]. BVAB1, BVAB2, and BVAB3 are three newly discovered bacteria from the *Clostridiales* order in the Vaginal Human Microbiome Project [17]. In BV, the amounts of these microorganisms—of relatively low virulence—are 100- to 1000-fold above the normal bacterial levels in a healthy vagina [18].

Current therapy for BV involves oral or intravaginal administration of metronidazole or clindamycin [19], but long-term follow-up suggests high recurrence rates [20] and, hence, the need for alternative therapeutic tools.

The human microbiota’s beneficial functions have led to the selection of bacterial strains, recognized as probiotics, with health-promoting activities to treat conditions where the microbiota composition or function is disturbed. In vaginal clinical conditions, *Lactobacillus* are the commonest bacteria used as probiotics, with the rationale based on the regulatory role played by lactobacilli in the vaginal ecosystem [21].

Oral and vaginal probiotic formulations have both been used for the treatment and prevention of BV. Probiotics that are taken orally are believed to reach the vaginal tract after being excreted from the rectum. Through vaginal administration, probiotic strains can replace unhealthy vaginal microbiota and occupy specific adhesion sites on the epithelial surface of the vagina directly, which consequently results in maintenance of a low pH and production of antimicrobial substances such as acids, hydrogen peroxide, and biosurfactants [22].

Given the importance of probiotics in promoting vaginal health, the aim of our study was to investigate the diversity and vaginal microbiota composition in asymptomatic women with BV, before and after the oral administration of a probiotic formulation, via 16s metagenomic analysis.

## 2. Materials and Methods

### 2.1. Study Design and Sample Collection

This was a prospective, observational pilot study with pre–post design, performed from 1 June 2022 to 31 December 2022 on a subset of patients from a larger study on vaginal microbiota composition in women with endometriosis. In particular, a total of 50 healthy reproductive-age women were enrolled amongst the patients attending the general gynecological outpatient consultation service of the University Hospital “Policlinico Umberto I” Rome for a routine consultation. Inclusion criteria were age between 20 and 40 years old and a recent Papanicolau test negative for malignancy or inflammation. Exclusion criteria were pre-menarche or menopause status, diabetes, neoplastic diseases, urinary or genital infections in the past 6 months, bowel and/or liver disorders, current treatment with oral contraceptive or progestins, prokinetics, antacids or proton pump inhibitors, sexual activity in the week before sampling, recent or current antibiotic treatment (oral or topical), as well as the use of probiotics and/or prebiotics for three months before the enrolment.

All study participants received the probiotic product CDS22-formula (also known under the tradenames Visbiome, De Simone, Vivomixx) as acid-resistant oral capsules (twice daily), containing 1.12 × 10^11^ live bacteria per capsule. The preparation includes eight live freeze-dried bacterial species (four strains of *lactobacilli* [*Lactobacillus paracasei* NCIMB 30439, *Lactobacillus plantarum* NCIMB 30437, *Lactobacillus acidophilus* NCIMB 30442, and *Lactobacillus helveticus* NCIMB 30440]; three strains of bifidobacteria [*Bifidobacterium animals* subsp. *lactis* NCIMB 30435, *B. animalis* subsp. *lactis* NCIMB 30436, and *Bifidobacterium breve* NCIMB 30441] and the *Streptococcus salivarius* subsp. *thermophilus* NCIMB 30438).

The patients were evaluated at baseline (*t*0) and after 2 months (*t*1) of probiotic administration; age, body mass index (BMI), age at menarche and smoking habits were recorded, and all women underwent a gynaecological examination; from each woman two vaginal swabs at *t*0 and *t*1 were collected, one was used for the preparation of Gram smears in order to calculate the Nugent score, and the other for the metagenomic analysis. The sampling was made at the time of ovulation, as detected by the ovulation test kit “Clearblue digital test” (Swiss Precision Diagnostics GmbH, Geneva, Switzerland); samples were immediately stored at −20 °C until further processing.

All study participants gave written informed consent to the study. The study was approved by the Umberto I University Hospital Ethics Committee (protocol n. 0751/2020, 19 October 2020) and conducted according to the principles expressed in the Declaration of Helsinki. This clinical trial was registered on https://clinicaltrials.gov/ (registration number NCT06592976, accessed on 19 September 2024).

### 2.2. Nugent Score Assessment and Calculation

Patients were assessed for BV by evaluating the Gram stain score of vaginal smears, according to the method developed by Nugent et al. [23].

### 2.3. Metagenomic Analysis of the Vaginal Microbiota Composition

#### 2.3.1. DNA Isolation and Next-Generation Sequencing

DNA isolation from vaginal samples was performed using QIAmp Blood&Tissue Mini kit (QIAGEN, Germantown, MD, USA), according to Manufacturer’s Instructions. DNA was quantified and its integrity was checked according to methods described by Filardo et al. [24].

#### 2.3.2. 16s rRNA Gene Amplification and Illumina MiSeq Sequencing

V3–V4 hypervariable regions of the 16s rRNA gene were amplified by two steps PCRs, and Illumina MiSeq Sequencing was carried out as previously described [24].

#### 2.3.3. Sequencing Data and Bioinformatic Analysis

Sequencing data and subsequent bioinformatic analysis were performed, after trimming of Illumina adaptor sequences and primers via cutadapt (version 4.9) [25], using the software framework QIIME 2 (version 2023.7) [26], as previously described [24].

Taxonomic assignment was performed against the novel reference tree Greengenes2 via the QIIME 2 plugin q2-greengenes2, according to the methodology described by McDonald et al., 2023 [27].

Alpha (via Shannon’s diversity and Faith’s Phylogenetic Diversity indexes) and beta (via weighted and unweighted UniFrac analysis) diversity comparisons were considered as biodiversity metrics, as previously described [24].

For the identification of taxonomic biomarkers, relative abundances based on all obtained reads were used. Differential taxonomic units between groups were identified using the linear discriminant analysis (LDA) coupled with effect size measurement (LEfSe) and the Analysis of Composition of Microbiomes (ANCOM), as previously described [28,29].

The classification of the vaginal microbiota in different community state types (CSTs) was performed by using the tool VAginaL community state typE Nearest CentroId classifier (VALENCIA), according to the methods described by France et al., 2020 [30].

### 2.4. Statistical Analysis

Parametric and non-parametric data were expressed as mean ± standard deviation (SD) and were analyzed by Student’s *t*-test, or by Mann–Whitney test for independent samples, and Wilcoxon Signed-Rank test for paired samples. The chi-squared test was used for assessment of association of frequencies among groups (Fisher’s exact test was used when any cell had expected values of <5). All statistical calculations were performed in Excel (version 2403, build 17425.20176 Click-to-Run, Microsoft, Redmond, WA, USA) via the add-in Real Statistics Resource Pack (https://real-statistics.com/free-download/real-statistics-resource-pack/ (accessed on 17 April 2024)). Relative abundances of taxa were expressed as means ± standard error of means (SEM), with alpha diversity indexes as median (IQR). Nonparametric *t-*test based on Monte Carlo permutations was used for alpha diversity comparisons, and Adonis was used for category comparisons of distance matrices, all calculated in QIIME 2 [26]. The alpha-correlation analysis between pH values and alpha diversity distances was performed via Pearson’s product–moment correlation. Bonferroni correction was used to correct for multiple hypothesis testing when needed. The single or multiple inference significance level was set at 5%.

## 3. Results

A total of 50 consecutive women were enrolled in the study; amongst them, 13 had a Nugent score of 7–8 and were included in the group of patients with asymptomatic BV, whereas 37 had a Nugent score of 1–3 and were included in the group of patients with a healthy genital microbiota (Table 1). All women with BV were asymptomatic. No statistically significant differences were observed in age, BMI, age at menarche and smoking habits between the two groups. After 2 months of probiotic supplementation, no side effects were reported in all the participants in the study.

### 3.1. Composition of the Vaginal Microbiota in the Study Population

The metagenomic analysis provided an average of 25,461 [median (Interquartile Range, IQR) 19,941 (14,787)] and 31,802 [22,156 (14,592)] paired-end Illumina reads in women with BV and women with a healthy vaginal microbiota at baseline (*t*0), respectively. After the removal of singletons and rare ASVs, an average number of 16.6 [18 (5)] and 8.9 [8 (6)] ASVs (*p =* 0.00016) was observed at *t*0 in women with BV and women with a healthy vaginal microbiota, respectively.

After 2 months of probiotic supplementation (*t*1), an average of 34,539 [21,398 (47,880)] and 30,754 [22,052 (11,450)] paired-end Illumina reads, and an average number of 11.9 [10 (8)] and 8.7 [7 (8)] ASVs (*p* = 0.03) were retrieved in women with BV and women with a healthy vaginal microbiota, respectively. Women with BV showed a statistically significant decrease in the average number of ASVs after probiotic supplementation [18 (5) at *t*0 and 10 (8) at *t*1, *p* = 0.012], while women with a healthy vaginal microbiota did not show any statistically significant difference [8 (6) at *t*0 and 7 (8) at *t*1, *p* = 0.07].

Overall, the lowest read was 3265, hence, the ASVs were randomly sub-sampled to this minimum read for diversity analysis, to avoid bias.

#### 3.1.1. Characterization of the Vaginal Microbiota at Baseline

**Figure 1:**
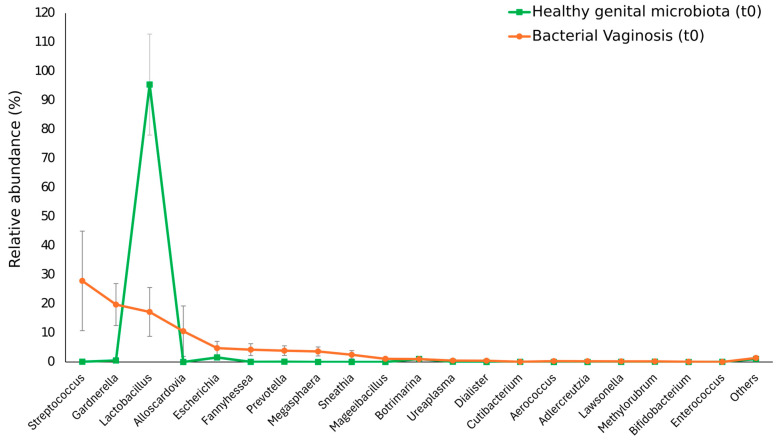
Vaginal microbiota composition in women with BV and women with a healthy vaginal microbiota at baseline. Only taxa with abundances greater than 0.01% in any sample were included in the graph.

At baseline (*t*0), significant differences in the vaginal microbiota composition were observed between women with BV, and women with a healthy vaginal microbiota. Concerning the CST classification (Table 1 and Figure 1), a significantly higher prevalence of CST-IV (69.2%) was observed in women with BV as compared to women with a healthy vaginal microbiota (5.4%, *p* < 0.00001). The CST-IV in women with BV included exclusively the subtypes CST-IV B (46.2%) and C1 (23.1%), characterized by the predominance of *Gardnerella vaginalis* and *Fannyhessea vaginae* (previously known as *Atopobium vaginae*), or *Streptococcus* spp., respectively. By contrast, a higher prevalence of CSTs characterized by *Lactobacillus* spp. predominance (94.6%), such as CST-I (*L. crispatus*, 73%), and CST-III (*L. iners*, 21.6%), was observed in women with a healthy vaginal microbiota as compared to women with BV (30.8%, *p* < 0.0001); interestingly, CST-II (*L. gasseri*) was only identified in women with BV (15.4% vs. 0% in women with a healthy vaginal microbiota, *p* < 0.05).

As for the bacterial composition at *t*0, shown in Table 2 and Figure 1, women with BV showed increased relative abundance, as compared to women with a healthy vaginal microbiota, for the bacterial genera *Streptococcus* spp. (27.9% vs. 0.1%, *p* < 0.01), *Gardnerella* spp. (19.8% vs. 0.5%, *p* < 0.0001), *Alloscardovia* spp. (10.6% vs. 0.003%, *p* < 0.01), *Fannyhessea* spp. (4.2% vs. 0.01%, *p* < 0.000001), *Prevotella* spp. (3.9% vs. 0.1%, *p* < 0.001), *Megasphaera* spp. (3.6% vs. 0.002%, *p* < 0.00001), *Sneathia* spp. (2.5% vs. 0.004%, *p* < 0.001), and *Aerococcus* spp. (0.3% vs. 0.001%, *p* < 0.00001). By contrast, women with a healthy vaginal microbiota had a significantly higher prevalence of *Lactobacillus* spp. (95.4%) than did women with BV (17.2%, *p* < 0.0000001) (Table 2).

#### 3.1.2. Characterization of the Vaginal Microbiota after 2 Months of Probiotic Supplementation

**Figure 2:**
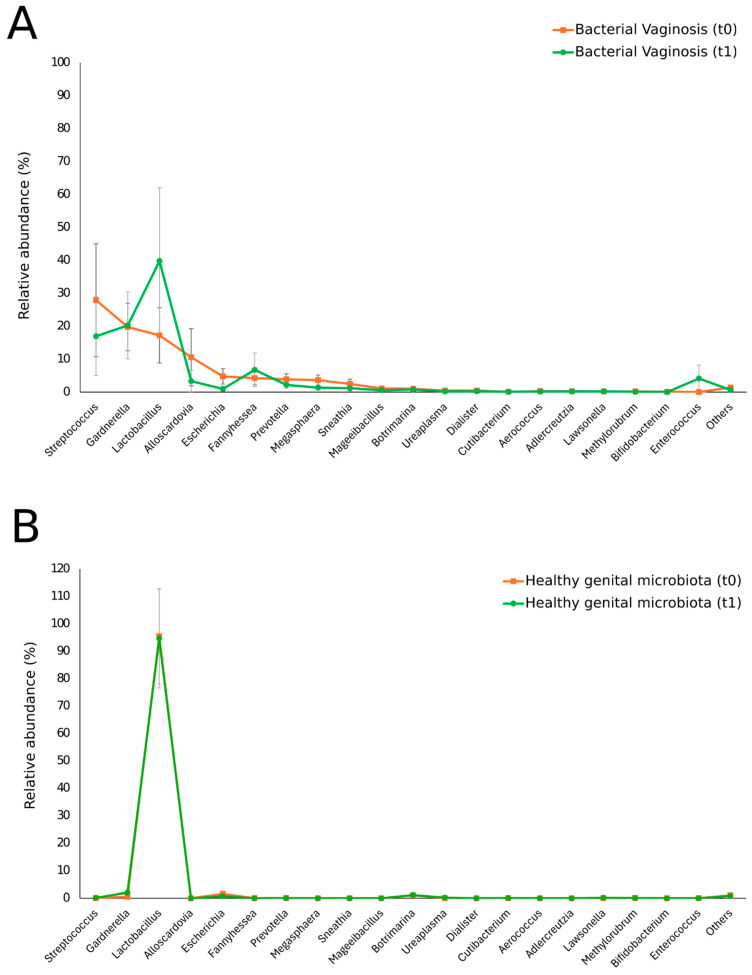
Vaginal microbiota composition in the study population before and after 2 months of probiotic supplementation. Women with asymptomatic BV (**A**) and women with a healthy vaginal microbiota (**B**), at *t*0 and *t*1. Only taxa with abundances greater than 0.01% in any sample were included in the graphs. All values are expressed as mean ± relative standard error.

After probiotic supplementation, women with BV showed a statistically significant decrease in the relative abundance of *Escherichia* spp. (4.8% at baseline vs. 1% at *t*1, *p* = 0.03) and *Megasphaera* spp. (3.6% at baseline vs. 1.3% at *t*1, *p* = 0.042), as shown in Table 3 and Figure 2A. Other relevant changes in the bacterial composition, albeit without reaching statistical significance, could also be observed in *Streptococcus* spp. (27.9% at baseline vs. 17% at *t*1), *Lactobacillus* spp. (17.2% at baseline vs. 39.8% at *t*1), *Alloscardovia* spp. (10.6% at baseline vs. 3.3% at *t*1) *Prevotella* spp. (3.9% at baseline vs. 2.2% at *t*1), and *Sneathia* spp. (2.5% at baseline vs. 1.2% at *t*1).

In women with a healthy vaginal microbiota at baseline, no differences were observed in the vaginal bacterial composition after 2 months of probiotic supplementation, as shown in Table 3 and Figure 2B. None of the BV or healthy women were classified in a different CST at *t*1 in comparison to *t*0.

### 3.2. Alpha- and Beta-Diversities Analysis

**Figure 3:**
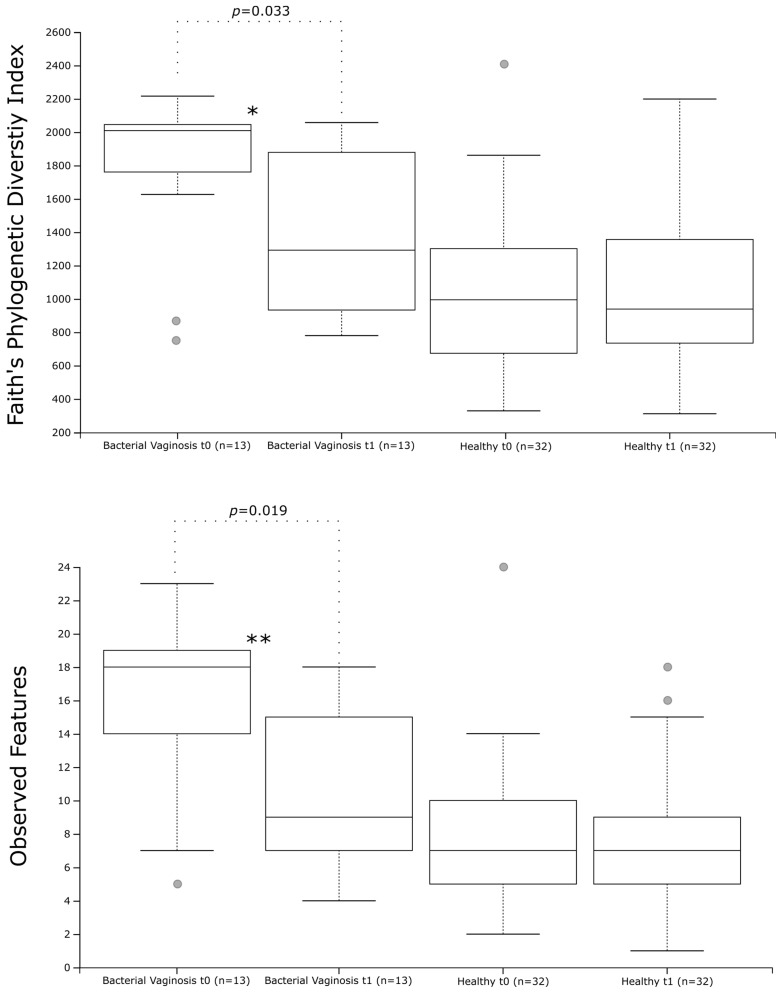
Comparison of the alpha-diversity of the vaginal microbiota in women with BV and women with a healthy vaginal microbiota, at baseline (*t*0) and after (*t*1) 2 months of probiotic supplementation. Faith’s phylogenetic diversity and observed features were used as measures of alpha-diversity within groups. The circles out of range represent the outliers. * *p* < 0.001 and ** *p* < 0.0001 vs. women with a healthy vaginal microbiota.

**Figure 4:**
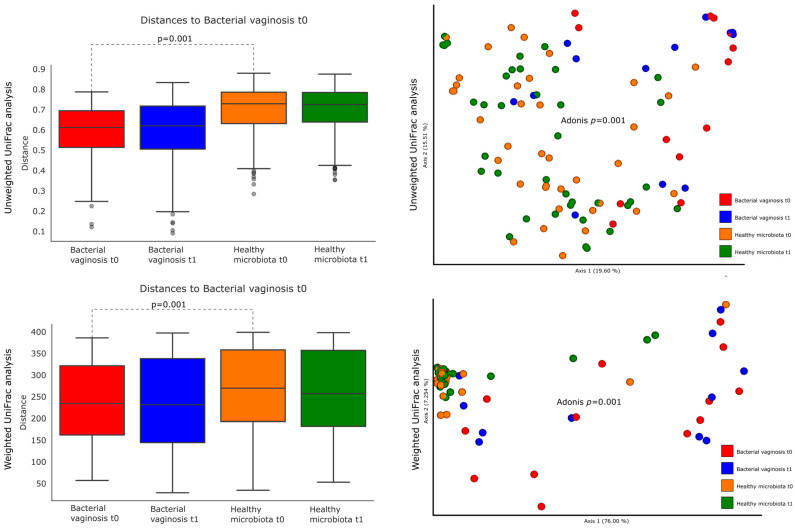
Comparison of the beta-diversity of the vaginal microbiota in women with BV and women with a healthy vaginal microbiota, at baseline (*t*0) and after (*t*1) 2 months of probiotic supplementation. On the left, the boxplot representations of within-group distances, and on the right the principal coordinate analysis (PCoA) plots, of unweighted and weighted UniFrac distance matrices, are illustrated. Each dot represents the vaginal bacterial community composition of one individual, and the groups were compared using Adonis for beta-diversity measures.

Comparing the diversity and richness of the vaginal microbiota at baseline, between women with BV and women with a healthy vaginal microbiota, as defined above, Faith’s phylogenetic diversity index showed a significantly higher diversity in the presence of BV (Figure 3, *p* = 0.0001). Similarly, the diversity measure observed features showed a higher richness of vaginal bacterial communities in women with BV than women with a healthy vaginal microbiota (Figure 3, *p* = 0.00006). Concerning the beta-diversity measures, a significant clustering of the bacterial communities found in the vaginal microenvironment of women with BV as compared to women with a healthy vaginal microbiota was observed in both unweighted (*p =* 0.001) and weighted (*p =* 0.001) UniFrac analysis, as shown in Figure 4.

After 2 months of probiotic supplementation, decreased diversity and richness of the bacterial communities of the vaginal microbiota was demonstrated exclusively in women who were categorized as having BV via the Nugent score at baseline. Specifically, a statistically significant lower diversity in women with BV at *t*1 than in women with BV at *t*0 was observed via both the Faith’s phylogenetic diversity (*p* = 0.03) and Observed features diversity index (*p* = 0.0189) (Figure 3). Conversely, no statistically significant difference before and after 2 months of probiotic supplementation were observed in women with a healthy vaginal microbiota (Figure 3). Both the unweighted and weighted UniFrac analyses did not highlight any statistically significant clustering of the bacterial communities of the vaginal microbiota, before and after 2 months of probiotic supplementation, in either women with BV or women with a healthy vaginal microbiota (Figure 4).

### 3.3. Identification of Specific Taxonomic Units as Potential Biomarkers

**Figure 5:**
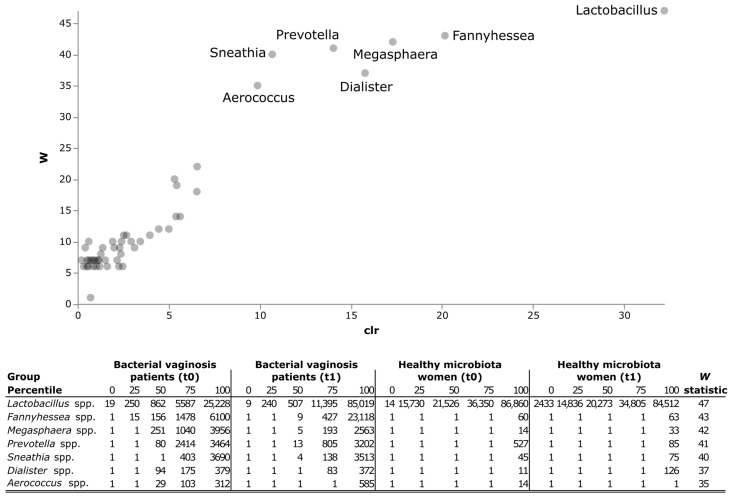
ANCOM test of the vaginal microbiota between women with BV and women with a healthy genital microenvironment, either at baseline or after 2 months of probiotic supplementation. ANCOM employs a heuristic strategy to declare taxa that are significantly differentially abundant, and, for any given taxon, the output *W* statistic represents the number of additive log ratio (ALR) transformed models, where the taxon is differentially abundant; hence, the larger the value of *W*, the more likely the taxon is differentially abundant.

As evidenced in Figure 5, The ANCOM test evidenced significant differences amongst the study groups for 7 bacterial genera; namely, *Lactobacillus* spp. (*W* statistic 47), *Fannyhessea* spp. (*W* statistic 43), *Megasphaera* spp. (*W* statistic 42), *Prevotella* spp. (*W* statistic 41), *Sneathia* spp. (*W* statistic 40), *Dialister* spp. (*W* statistic 37), and *Aerococcus* spp. (*W* statistic 35). In particular, *Lactobacillus* spp. was prevalent in women with a healthy vaginal microbiota, either before or after the 2 months of probiotic supplementation. Interestingly, after the intervention, women with BV also showed an increase in the prevalence of *Lactobacillus* spp. By contrast, the other bacterial genera identified by the ANCOM analysis were mostly present in the vaginal microbiota of women with BV, with *Pretovella* spp., *Sneathia* spp., *Dialister* spp., and *Aerococcus* spp. abundant either at baseline or after 2 months of probiotic supplementation. Only *Megasphaera* spp. decreased, whereas *Fannyhessea* spp. increased, in women with BV after probiotic supplementation.

Concerning the LEfSe analysis, it did not identify any statistically significant association between taxonomic units at any level in women with BV or women with a healthy vaginal microbiota, either before or after the 2 months of probiotic supplementation.

## 4. Discussion

The main result of our pilot study lies in the significant improvement of the vaginal microbiota in women with asymptomatic BV after 2 months of supplementation with a multi-strain oral probiotic (2.2 × 10^11^ CFU per day of *L. paracasei*, *L. plantarum*, *L. acidophilus*, *L. helveticus*, two variants of *B. animals* subsp. *lactis*, *B. breve* and *S. salivarius* subsp. *thermophilus*). In fact, the diversity and richness of the bacterial communities in the vaginal microenvironment of this group were significantly reduced, as evidenced by measures of alpha-diversity; more importantly, some bacterial genera typically associated to a condition of genital dysbiosis, such as *Megasphaera* spp., significantly decreased, while at the same time, *Lactobacillus* spp. doubled. In women with a healthy vaginal microenvironment, supplementation with the oral probiotic did not influence the genital microbiota composition. It is important to underline the significant reduction in the relative abundance of *Escherichia* spp. in women with BV after probiotic administration. Indeed, *Escherichia coli* represents one of the main pathogens involved in urinary infection. A recent paper investigating the effectiveness of prophylactic oral and vaginal probiotics in the prevention of recurrent UTIs in premenopausal women reported that vaginal *E. coli* counts were significantly reduced in the oral probiotic group compared with the placebo group [31]. Notably, Gupta and colleagues used the same oral probiotic used in the present study.

Therefore, our preliminary results suggest the multi-strain oral probiotic as a beneficial treatment specifically targeting the dysbiotic vaginal microenvironment, underlining the role of a healthy intestinal microbiota in vaginal health. This is particularly important from a clinical perspective, since a preventive approach based on the administration of an oral probiotic, especially in women with asymptomatic BV, may help in reducing the risk for urogenital infections or reproductive complications, as well as improving the overall quality of life. The bacteria typically involved in BV can migrate to the upper genital tract, reaching the uterus and fallopian tubes and causing pelvic inflammatory disease. In up to 10–30% of pregnant women with BV, this imbalance may be associated with preterm delivery, often (up to 70% worldwide) accompanied by perinatal mortality. BV also increases the risk for acquiring sexually transmitted infections, such as HPV, HSV, and HIV; as well as bacterial pathogens including *Neisseria gonorrhoeae* and *Chlamydia trachomatis*, further contributing to severe pathologies such as cervical cancer and infertility [32,33].

Few studies have been performed over the years showing the influence of oral probiotic products, including different species of *Lactobacillus*, on the vaginal microbiota homeostasis in women with BV. However, all of them did not investigate the full composition of the vaginal microbiome via 16s rDNA sequencing and, instead, evaluated the condition of the genital microbiota via the Nugent score, or the quantification of *Lactobacillus* spp. or *G. vaginalis* via either real-time PCR or cytologic smear analyses [33,34,35,36].

As the only study based on the metagenomic analysis of 16s rDNA, Ansari et al. supported the beneficial effects of oral probiotics on the vaginal microbiota, alongside our study, showing that the oral administration of a combination of *Lactobacillus acidophilus*, *L. rhamnosus*, and *L. reuteri* (10^10^ CFU of total bacterial strains per day) improved vaginal dysbiosis after 6 weeks of treatment in 36 asymptomatic women; in particular, they showed higher colonization of the vagina with the same lactobacilli from the probiotic formulation, via qRT PCR; supporting the hypothesis of a translocation of bacterial strains from the gut to the vaginal microenvironment [37]. By contrast, in our study the same probiotic strains could not be identified in the vaginal microbiota via 16s metagenomic analysis. This suggests the interesting hypothesis of an indirect effect of our probiotic strains via a complex interplay between their metabolites and the genital ecosystem, although we cannot exclude that the probiotic lactobacilli could be present in minimal quantities, undetectable via metagenomic analysis.

Our pilot study possesses important strengths that improve the robustness of our data. In particular, the use of 16s rRNA gene sequencing has allowed comprehensive profiling of the human microbiota composition up to the identification of specific microbial community types, reaching a deeper understanding of the importance of microbiota in the etiopathogenesis of BV. Indeed, to date, most studies focused on microscopy, bacterial culturing, and clinical criteria, defining only a state of genital microbiota imbalance. Furthermore, in our study vaginal swabs were collected at the time of ovulation, as detected by the ovulation test “Clearblue digital test kit”, either before or after the oral probiotic administration, reducing the potential bias due to physiological variations in the host hormonal state, since it is known that female sex hormones can modulate the genital ecosystem [38]. Conversely, the main limitation of our work lies in the small group of women with asymptomatic BV, due to the difficulties in identifying women with this condition, since the Nugent test is not widely applied as screening approach in the general population. A further weakness consists of the inability of 16s metagenomic analysis to identify the presence of low-abundance bacteria in the vaginal microbiota.

## 5. Conclusions

Overall, our preliminary results suggest the multi-strain oral probiotic as a beneficial treatment specifically targeting the dysbiotic vaginal microenvironment. However, important challenges still need to be solved; in particular, the type and quantity of live bacteria included in the probiotic formulation, the type of formulation (tablets, capsules, packets, etc.) and, importantly, the duration and frequency of treatment must be evaluated in a large number of subjects. The route of administration is also a challenge; from a regulatory perspective, oral probiotics are generally recognized as safe by many health authorities and their use is widespread in various forms, from supplements to fortified food products [39,40]. This contrasts with vaginal probiotics, which can be subject to more stringent regulations given their route of administration [41]. The regulation of probiotics differs between countries: there is no universally agreed framework. In the European Union, probiotics and food supplements are regulated under the Food Products Directive and Regulation (regulation 178/2002/EC; directive 2000/13/EU). In this scenario, oral probiotics may be considered as a better alternative to vaginal probiotics, especially considering the regulatory hurdles in some countries and patient compliance [42].

In the future, larger studies employing more advanced multi-omics analyses, alongside DNA sequencing, will be necessary to clearly describe the etiopathogenetic relationships between resident microorganisms and genital pathologies, including BV; leading to the discovery of individual microbial profiles that would enable a precision-medicine approach.

## Figures and Tables

**Table 1:** General characteristics of study population.

	Asymptomatic BV Patients (*n* = 13)	Women with Healthy Vaginal Microbiota (*n* = 37)
Age (mean ± SD)	28.6 ± 3.1	25 ± 5.7
BMI (mean ± SD)	23.2 ± 3.4	22.7 ± 4.5
Age at menarche (mean ± SD)	11.9 ± 1.1	12.1 ± 1.5
Smoke [*n* (%)]	4 (26.7)	8 (21.6)
Nugent score (mean ± SD)	7.5 ± 0.5	2.25 ± 1.33
**Vaginal Microbiota Classification [*n* (%)]**		
CST-I (*L. crispatus*)	1 (7.7)	27 (73.0)
CST-II (*L. gasseri*)	2 (15.4)	0 (0)
CST-III (*L. iners*)	1 (7.7)	8 (21.6)
CST-IV B (*G. vaginalis*, *F. vaginae*)	6 (46.2)	2 (5.4)
CST-IV C (*Streptococcus* spp.)	3 (23.1)	0

BMI, body mass index; SD, standard deviation; CST, community state type.

**Table 2:** Baseline bacterial composition at the genus level.

	Asymptomatic BV (*n* = 13)	Healthy Microbiota (*n* = 37)	*p* Values
*Streptococcus*	27.9 ± 17.1	0.1 ± 0.03	0.006
*Gardnerella*	19.7 ± 7.2	0.5 ± 0.4	0.00006
*Lactobacillus*	17.2 ± 8.4	95.4 ± 17.4	0.00000005
*Alloscardovia*	10.6 ± 8.7	0.003 ± 0.002	0.002
*Escherichia*	4.8 ± 2.3	1.6 ± 0.8	NS
*Fannyhessea*	4.2 ± 2.0	0.01 ± 0.01	0.0000001
*Prevotella*	3.9 ± 1.7	0.1 ± 0.1	0.0002
*Megasphaera*	3.6 ± 1.6	0.002 ± 0.001	0.000003
*Sneathia*	2.5 ± 1.4	0.004 ± 0.004	0.0004
*Mageeibacillus*	1.1 ± 0.6	0.006 ± 0.005	NS
*Botrimarina*	1.0 ± 0.8	1.0 ± 0.3	NS
*Ureaplasma*	0.5 ± 0.4	0.1 ± 0.1	NS
*Dialister*	0.4 ± 0.2	0.001 ± 0.001	NS
*Cutibacterium*	0.1 ± 0.1	0.02 ± 0.01	NS
*Aerococcus*	0.3 ± 0.1	0.001 ± 0.001	0.000003
*Adlercreutzia*	0.3 ± 0.1	0.0 ± 0.0	NS
*Lawsonella*	0.2 ± 0.2	0.05 ± 0.03	NS
*Methylorubrum*	0.2 ± 0.1	0.1 ± 0.03	NS
*Bifidobacterium*	0.1 ± 0.05	0.003 ± 0.004	NS
*Enterococcus*	0.01 ± 0.01	0.01 ± 0.01	NS
Others	1.4 ± 0.4	1.1 ± 0.3	NS

NS, not significant.

**Table 3:** Bacterial composition at the genus level after 2 months of probiotic supplementation.

	Asymptomatic BV (*n* = 13)		Healthy Microbiota (*n* = 37)	
	*t*0	*t*1	*t*0	*t*1
*Streptococcus*	27.9 ± 17.1	17.0 ± 11.9	0.1 ± 0.03	0.1 ± 0.1
*Gardnerella*	19.7 ± 7.2	20.3 ± 10.1	0.5 ± 0.4	2.0 ± 1.6
*Lactobacillus*	17.2 ± 8.4	39.8 ± 22.3	95.4 ± 17.4	94.6 ± 18.1
*Alloscardovia*	10.6 ± 8.7	3.3 ± 3.3	0.003 ± 0.002	0.0 ± 0.0
*Escherichia*	4.8 * ± 2.3	1.0 ± 0.7	1.6 ± 0.8	0.7 ± 0.3
*Fannyhessea*	4.2 ± 2.0	6.8 ± 5.1	0.01 ± 0.01	0.01 ± 0.01
*Prevotella*	3.9 ± 1.7	2.2 ± 1.1	0.1 ± 0.1	0.02 ± 0.01
*Megasphaera*	3.6 * ± 1.6	1.3 ± 0.7	0.002 ± 0.001	0.004 ± 0.003
*Sneathia*	2.5 ± 1.4	1.2 ± 0.8	0.004 ± 0.004	0.008 ± 0.007
*Mageeibacillus*	1.1 ± 0.6	0.5 ± 0.3	0.006 ± 0.005	0.005 ± 0.003
*Botrimarina*	1.0 ± 0.8	0.8 ± 0.3	1.0 ± 0.3	1.1 ± 0.3
*Ureaplasma*	0.5 ± 0.4	0.2 ± 0.2	0.1 ± 0.1	0.2 ± 0.2
*Dialister*	0.4 ± 0.2	0.2 ± 0.1	0.001 ± 0.001	0.0003 ± 0.0003
*Cutibacterium*	0.1 ± 0.1	0.1 ± 0.04	0.02 ± 0.01	0.1 ± 0.03
*Aerococcus*	0.3 ± 0.1	0.2 ± 0.1	0.001 ± 0.001	0.0 ± 0.0
*Adlercreutzia*	0.3 ± 0.1	0.2 ± 0.1	0.0 ± 0.0	0.0 ± 0.0
*Lawsonella*	0.2 ± 0.2	0.2 ± 0.2	0.05 ± 0.03	0.1 ± 0.1
*Methylorubrum*	0.2 ± 0.1	0.1 ± 0.1	0.1 ± 0.03	0.1 ± 0.04
*Bifidobacterium*	0.1 ± 0.05	0 ± 0	0.003 ± 0.004	0.003 ± 0.004
*Enterococcus*	0.01 ± 0.01	4.1 ± 4.1	0.01 ± 0.01	0.002 ± 0.002
Others	1.4 ± 0.4	0.6 ± 0.2	1.1 ± 0.3	0.8 ± 0.2

*t*0, before, and *t*1, after 2 months of probiotic supplementation; * *p* < 0.05 vs. *t*1 in women with BV.

## Data Availability

The raw 16 rDNA sequences were deposited with links to BioProject accession number PRJNA1137153 in the Sequence Read Archive (SRA) repository (https://www.ncbi.nlm.nih.gov/, accessed on 18 July 2024).
